# Anticancer actions of carnosine in cellular models of prostate cancer

**DOI:** 10.1111/jcmm.18061

**Published:** 2023-11-29

**Authors:** K. Habra, J. R. D. Pearson, P. Le Vu, C. Puig‐Saenz, M. J. Cripps, M. A. Khan, M. D. Turner, C. Sale, S. E. B. McArdle

**Affiliations:** ^1^ John van Geest Cancer Research Centre, School of Science and Technology Nottingham Trent University Nottingham UK; ^2^ Chemistry Department, School of Science and Technology Nottingham Trent University Nottingham UK; ^3^ Centre for Systems Health and integrated Metabolic Research (SHiMR), School of Science and Technology Nottingham Trent University Nottingham UK; ^4^ Centre for Diabetes, Chronic Diseases, and Ageing, School of Science and Technology Nottingham Trent University Nottingham UK; ^5^ Department of Urology University Hospitals of Leicester NHS Trust Leicester UK; ^6^ Institute of Sport, Manchester Metropolitan University Manchester UK

**Keywords:** cancer, carnosine, prostate, SIRT3, sustained release, tumour

## Abstract

Treatments for organ‐confined prostate cancer include external beam radiation therapy, radical prostatectomy, radiotherapy/brachytherapy, cryoablation and high‐intensity focused ultrasound. None of these are cancer‐specific and are commonly accompanied by side effects, including urinary incontinence and erectile dysfunction. Moreover, subsequent surgical treatments following biochemical recurrence after these interventions are either limited or affected by the scarring present in the surrounding tissue. Carnosine (β‐alanyl‐L‐histidine) is a histidine‐containing naturally occurring dipeptide which has been shown to have an anti‐tumorigenic role without any detrimental effect on healthy cells; however, its effect on prostate cancer cells has never been investigated. In this study, we investigated the effect of carnosine on cell proliferation and metabolism in both a primary cultured androgen‐resistant human prostate cancer cell line, PC346Flu1 and murine TRAMP‐C1 cells. Our results show that carnosine has a significant dose‐dependent inhibitory effect in vitro on the proliferation of both human (PC346Flu1) and murine (TRAMP‐C1) prostate cancer cells, which was confirmed in 3D‐models of the same cells. Carnosine was also shown to decrease adenosine triphosphate content and reactive species which might have been caused in part by the increase in SIRT3 also shown after carnosine treatment. These encouraging results support the need for further human in vivo work to determine the potential use of carnosine, either alone or, most likely, as an adjunct therapy to surgical or other conventional treatments.

## 1 INTRODUCTION

Prostate cancer is the most common cancer and the second leading cause of cancer death amongst men.^1^ Five‐year survival rate for patients with localized disease is almost 100%, but this number drops to 28% for patients diagnosed with metastatic disease.^2^ Treatment usually involves prostatectomy, radiation therapy, chemotherapy and hormone deprivation therapy. Many patients with intermediate to high‐risk localized prostate cancer will be offered radical prostatectomy (RP), brachytherapy or external beam radiotherapy depending on their age, life expectancy, prostate‐specific antigen (PSA) level, Gleason score, MRI staging and bone scan findings.^3^

Radical prostatectomy has not been encouraged in the past for patients with locally advanced diseases. Interestingly, Yossepowitch et al. showed that many patients categorized as ‘high risk’ have pathologically organ‐confined cancer and a nonuniform prognosis after RP.^3^ High‐grade prostate cancer patients are not regarded as good candidates for RP due to the high incidence of cancer‐positive pelvic lymph nodes and poor long‐term survival rates.^4^, ^5^ External beam radiotherapy uses high‐energy beams, such as x‐rays or protons, generated by a linear accelerator and directed towards the prostate gland to kill the cancer cells. Brachytherapy involves implanting small radioactive pellets into the prostate gland to deliver cancer‐damaging radiation directly to the tumours. Chen et al. have compared these methods to treat localized prostate cancer.^6^ They showed that patients who chose RP had a decreased risk of overall and cancer‐specific mortality and an improved 5‐ and 10‐year overall survival and cause‐specific survival. However, RP carries significant side effects such as urinary incontinence and erectile dysfunction. Additionally, many men with intermediate risk localized prostate cancer refuse immediate RP or radical radiotherapy/brachytherapy. Radiation therapy and chemotherapy are also associated with adverse events due to their systemic toxicity, meaning that normal noncancerous cells are also affected by therapy. As a result, novel cancer‐targeting therapeutics with little to no side effects need to be employed to prevent deleterious side effects and prevent cessation of patient therapy.

Carnosine (β‐alanyl‐L‐histidine) is a naturally occurring dipeptide composed of the amino acids β‐alanine and L‐histidine, which was originally discovered in skeletal muscle where it is found at higher concentrations than in any other tissues except brain.^7^, ^8^ The biological role of carnosine has been studied in relation to its ability to buffer intramuscular pH and support exercise capacity, as well as its antioxidant, anti‐inflammatory, anti‐glycation and anti‐chelating properties in healthy cells.^7^, ^8^ Carnosine has, however, also been shown to inhibit the proliferation of many types of cancer cells by affecting the production of reactive oxygen species.^9^, ^10^ Glioblastoma cells co‐cultured with normal fibroblasts and carnosine were shown to be the only cells affected by carnosine treatment, demonstrating the cancer‐specific effect of carnosine.^11^ Carnosine has demonstrated a protective role towards normal healthy cells,^12^ for example increasing the number of in vitro passages of primary human fibroblasts and rejuvenation of already senescent cells.^13^

Herein we investigated the use of carnosine as a potential adjunct to standard therapy for organ‐confined prostate cancer. Its minimal side effects and lack of toxicity would allow its use prior to organ confined prostate cancer standard therapy, thereby avoiding associated side effects and maintaining the possibility to use these later.^14^ The only reported side effect is paresthesia after a high intolerable single dose of 10 mg kg^−1^ which corresponds to an average of 800 mg of β‐alanine treatment,^15^ whilst exerting a wide range of potential beneficial biological effects in other tissues, including the brain, heart, pancreas and kidney.^16^ The effect of carnosine on such a metabolically unique cancer has never been assessed and, therefore, we set out to determine the effect of carnosine on a primary androgen resistant prostate cancer cell line (PC346Flu1) and a murine prostate cancer cell line (TRAMP‐C1) in vitro to investigate its mechanisms of action.

## 2 MATERIALS AND METHODS

### 2.1 Cell culture

The PC346C cell line was established from a transurethral resection of a primary tumour expressing the wild‐type androgen receptor (AR).^17^ PC346Flu1 cells were derived by culture of PC346C cells in steroid‐stripped medium supplemented with the anti‐androgen hydroxyflutamide. As a result, PC346Flu1 strongly upregulated AR expression, thereby becoming androgen independent, a phenomenon known to happen to prostate cancer patients who are treated with long‐term androgen ablation and whose tumour initially responds but then relapses within 1 to 3 years as an incurable hormone‐refractory condition.^17^ The cells were a generous gift from Professor van Weerdan, Rotterdam, The Netherlands. Cells were cultured in DMEM/F12 (Lonza) supplemented with 0.001% BSA (Albumin Fraction V from bovine serum; Sigma‐Aldrich), 2% charcoal stripped fetal bovine serum, 10 ng/mL EGF (Sigma‐Aldrich), 1% Insulin‐Transferrin‐Selenium (Gibco), 0.5 μg/mL hydrocortisone (Sigma‐Aldrich), 0.6 ng/mL triiodothyronine (Sigma‐Aldrich), 0.1 mM phosphoethanolamine (Sigma‐Aldrich), 50 ng/mL cholera toxin (Sigma‐Aldrich) and 100 ng/mL fibronectin (Sigma‐Aldrich), 20 μg/mL fetuin (ICN Biomedicals Inc.). TRAMP‐C1 cells were a generous gift from Dr. Matteo Bellone, San Raffaele Scientific Institute, Milan, Italy. This cell line was derived from prostatic epithelial cells from transgenic adenocarcinoma of the mouse prostate (TRAMP) model. Cells were grown in DMEM with 10% FBS and 2 mM L‐glutamine. DU 145 (HTB‐81) and PC3 cells were obtained from the ATCC; DU 145 cells were grown in MEM with Earle's salts and L‐glutamine (Corning) supplemented with 10% FBS, 1 mM sodium pyruvate (Lonza) and 1X MEM non‐essential amino acid solution (Sigma‐Aldrich), and PC3 cells were grown in F‐12 K (Corning) supplemented with 10% FBS and 2 mM L‐glutamine.

### 2.2 MTT assay for assessment of mitochondrial function

To assess the effect of carnosine on prostate cancer cells (murine and human), an initial MTT assay was performed. For each cell line, the experiment was designed with three biological replicates. Cells were seeded in 96‐well plates to reach 70%–80% confluency at the beginning of carnosine treatment. After the cells were incubated overnight to adhere, they were treated with a series of carnosine concentrations (0–300 mM for all cells besides PC346Flu1, which was treated with 0–1000 mM of carnosine). After 24 h of carnosine treatment for TRAMP‐C1 and PC346Flu1 and 48 h for DU 145 and PC3, cell viability was measured by MTT assay. 10 μL of MTT was added to each well at 5 mg/mL, and the plates were incubated in the dark at 37°C for 2 h. The medium was removed by aspiration and the insoluble formazan dye was solubilized in 100 μL of dimethyl sulfoxide (DMSO). The absorbance was measured at 570 nm using a CLARIOstar Plus microplate reader.

### 2.3 Detection of reactive species

PC346Flu1 cells were cultured in standard tissue culture media. Carnosine was added (0, 10, 100, 200 and 300 mM) and incubated for 24 h. Cells were washed three times in Krebs‐Ringer buffer (KRB), then 20 μM 2′,7′‐dichlorofluorescin diacetate (DCFDA) loaded for 1 h. Radical species detection was measured via fluorescence, with excitation at 495 nm and emission at 530 nm. RS are expressed as percentage changes relative to untreated control cells.

### 2.4 Detection of mitochondria and intracellular RS co‐localisation

PC346Flu1 cells were seeded to reach 70%–80% confluency. After cells were attached, medium containing 100 mM carnosine was prepared and applied to cells. After 0 and 3 h, the carnosine was removed, and cells were washed with PBS. Cells were then treated with 10 μM H_2_DCFDA (TOCRIS) and 200 nM MitoMark Red I (TOCRIS) for 30 min at 37°C. After treatment, cells were washed with PBS and then imaged with a fluorescence microscope, and changes in the co‐localisation of mitochondria with RS were observed visually.

### 2.5 Detection of *SIRT3* expression via RT‐qPCR

Cells were treated with 100 mM (TRAMP‐C1, DU 145 and PC3), 200 mM (TRAMP‐C1), 250 mM and 400 mM (PC346Flu1) of carnosine for 24 h and then pelleted. Total RNA was extracted using the RNeasy Mini Extraction Kit (QIAGEN) according to the manufacturer's protocol and the concentration of extracted RNA was measured on a NanoDrop spectrophotometer (Thermo Fisher). To synthesize cDNA, 2 μg of RNA in nuclease‐free water (final volume 9 μL) and 1 μL of oligo dT (Promega) were heated at 70°C for 5 min. A mix containing 5 μL of RT buffer (Promega), 1 μL of Reverse Transcriptase enzyme (Promega), 0.7 μL of RNasin (Promega), 1 μL of dNTPs (Promega) and 7.3 μL of nuclease‐free water were then added to the reaction and incubated at 40°C for 1 h. The reaction was heated at 95°C for 5 min and frozen immediately at −20°C.

The RT‐qPCR reaction was performed in a final volume of 13 μL by mixing 1 μL of cDNA, 6.25 μL of iTaq Universal SYBR Green supermix (Bio‐Rad), 0.5 μL of forward and reverse primer (10 pM) and 4.75 μL of nuclease‐free water. The relative expression was measured using the 2^−ΔCt^ method with two housekeeping genes (*HPRT* and *GUSB*). Cycling conditions were as follows: initial denaturation at 95°C for 5 minutes followed by 35 cycles of denaturation at 95°C for 10 seconds, annealing at 58°C for 15 seconds and extension at 72°C for 20 seconds.

**Table:** 

Gene	Forward primer	Reverse primer
hSIRT3	GAAACTGGGAAGCTTGATG	CTTGTCAGAATTGGGATGTG
hHPRT	TGACACTGGCAAAACAATGCA	GGTCCTTTTCACCAGCAAGCT
hGUSB	ACTGAACAGTCACCGAC	AAACATTGTGACTTGGCTAC

### 2.6 Detection of SIRT3 expression via western blot

PC346Flu1 cells were either left untreated (control) or treated with 250 mM or 400 mM carnosine dissolved in cell culture medium. After 24 h of carnosine treatment cells were lysed using radioimmunoprecipitation assay (RIPA) buffer with protease inhibitor (Sigma‐Aldrich), vigorously vortexed then placed on ice every 10 min for a 30‐min period. The mixture was then centrifuged at 14,000 × ***g*** for 15 min at 4°C to separate the cellular debris from the lysate. The concentration of protein within the lysate was quantified using a bicinchoninic acid (BCA) assay. For western blotting analysis, an equal amount of protein (20 μg) was loaded per well. Proteins were separated using SDS‐PAGE and then transferred to a nitrocellulose membrane. Membranes were then blocked using a 5% (w/v) milk solution in TBS‐T. After blocking, membranes were probed with antibodies against SIRT3 (mouse monoclonal from Santa Cruz Biotechnology) and vinculin (rabbit monoclonal from Abcam). After exposure to primary antibodies, membranes were washed and probed with HRP‐conjugated horse anti‐mouse and goat anti‐rabbit IgG antibodies (Cell Signaling Technology). Chemiluminescent signals were then detected using Clarity Western ECL substrate (Bio‐Rad) and imaged using a Syngene G:BOX station with GeneSys image acquisition software (Syngene). ImageJ software (NIH) was then used to quantify the band intensity.

### 2.7 Live‐cell imaging (proliferation and cytotoxicity)

To study the effect of carnosine on proliferation, the Incucyte Live‐Cell Analysis System (Essen BioScience) was used. Proliferation and cytotoxicity tests were carried out on TRAMP‐C1 and PC346Flu1 cells after applying different carnosine concentrations (0, 50, 100, 150, 200, 300 and 400 mM for PC346Flu1 cells; 0, 25, 50, 75, 100, 125, 150, 200 and 300 mM for TRAMP‐C1 cells) and using five replicates per treatment group. Cells were seeded in a 96‐well plate at a density of 5 × 10^3^ (PC346Flu1) and 1 × 10^3^ (TRAMP‐C1) cells per well and left for 24 h to adhere. Carnosine was added accordingly in the presence of 250 nM of Incucyte Cytotox Red Dye (Essen BioScience) for counting dead cells. This cyanine nucleic acid dye permeated cells with compromised cell membranes. The images were taken with a 10× objective every hour over 48 h. For both proliferation and cytotoxicity experiments, cell confluence was determined by using the Incucyte Base Analysis Software (version 2020B).

### 2.8 Sustained release simulation of carnosine on single spheroids

Each well of a green‐coded 96‐well round base plate for suspension (Sarstedt) was rinsed with 50 μL Anti‐Adherence Rinsing Solution (STEMCELL Technologies). After 15 min, all wells were washed with 50 μL of serum‐free medium. The PC346Flu1 and TRAMP‐C1 monolayers of cells were detached with trypsin (Sigma‐Aldrich) to generate a single‐cell suspension. The cells were counted and seeded at 400 cells per well in 100 μL of complete culture medium (details in Section 2.1) and spheroids were formed by centrifuging the plates at 2700 × g for 10 min. The plate was incubated at 37°C, 5% CO_2_ in a humidified incubator, and carnosine was added every other day at different concentrations ranging from 0 to 150 mM.^18^ The experiment was designed with up to four technical replicates per treatment group. The images were collected after 7 days, with the spheroid area being measured manually using the Incucyte Spheroid Analysis Software Module's measuring tool.

### 2.9 Seahorse XF Real‐Time ATP Rate Assay

Extracellular flux analysis was completed by the XF24 Analyzer (Seahorse Bioscience). For optimisation, four different cell densities were seeded into an XF24 cell culture microplate (100, 80, 60 and 40 × 10^3^ cells per well) and incubated for 24 h. All wells were seeded with cells except A1, B4, C3 and D6, which were left as a background. The sensor cartridge was hydrated by adding 1 mL of the Agilent Seahorse XF 24 calibrant solution to each well of the utility plate, then the plate was placed in a non‐CO_2_ 37°C incubator overnight. On the day of the assay, the medium was supplemented according to the manufacturer's guidelines.^19^ The cell culture microplate was prepared in advance by seeding the optimized density for each cell line in 250 μL and incubating for 24 h. 150 μL of the media was aspirated from each well, then 900 μL of assay media was added. After 15 min, 750 μL was aspirated from each well and 250 μL of assay media was added to get the starting well volume of 500 μL assay medium. On the day of the experiment, carnosine was diluted with the same medium to the following concentrations: 0, 50, 100 and 150 mM, with 56 μL in x10 concentration added to port A to be added to the starting well volume 500 μL assay medium. 62 μL of Oligomycin were added in port B and 69 μL of Rotenone with Antimycin in port C. The plate was placed in a non‐CO_2_ 37°C incubator for 45 min prior to the assay. The hydro booster plate was removed, and a constant volume of each compound was injected in the required port as mentioned above to get the x10 concentration. The XF Real‐Time ATP Rate Assay was run and analysed using XF software and the data analysis was completed automatically from the Agilent Seahorse XF Real‐Time ATP Rate Assay Report Generator. The Seahorse experiment as described above was repeated two more times for each cell line giving three biological replicates.

### 2.10 Statistical analysis

Data were analysed using GraphPad Prism (version 8) software. Student's *t*‐test and one‐way anova tests with post hoc Tukey's multiple comparisons were used for multigroup comparisons. *p* < 0.05 was considered statistically significant and all data were presented as mean ± SD.

## 3 RESULTS

### 3.1 Carnosine reduces the growth and induces the death of TRAMP‐C1 and PC346Flu1 cells in a dose‐dependent manner

**FIGURE 1:**
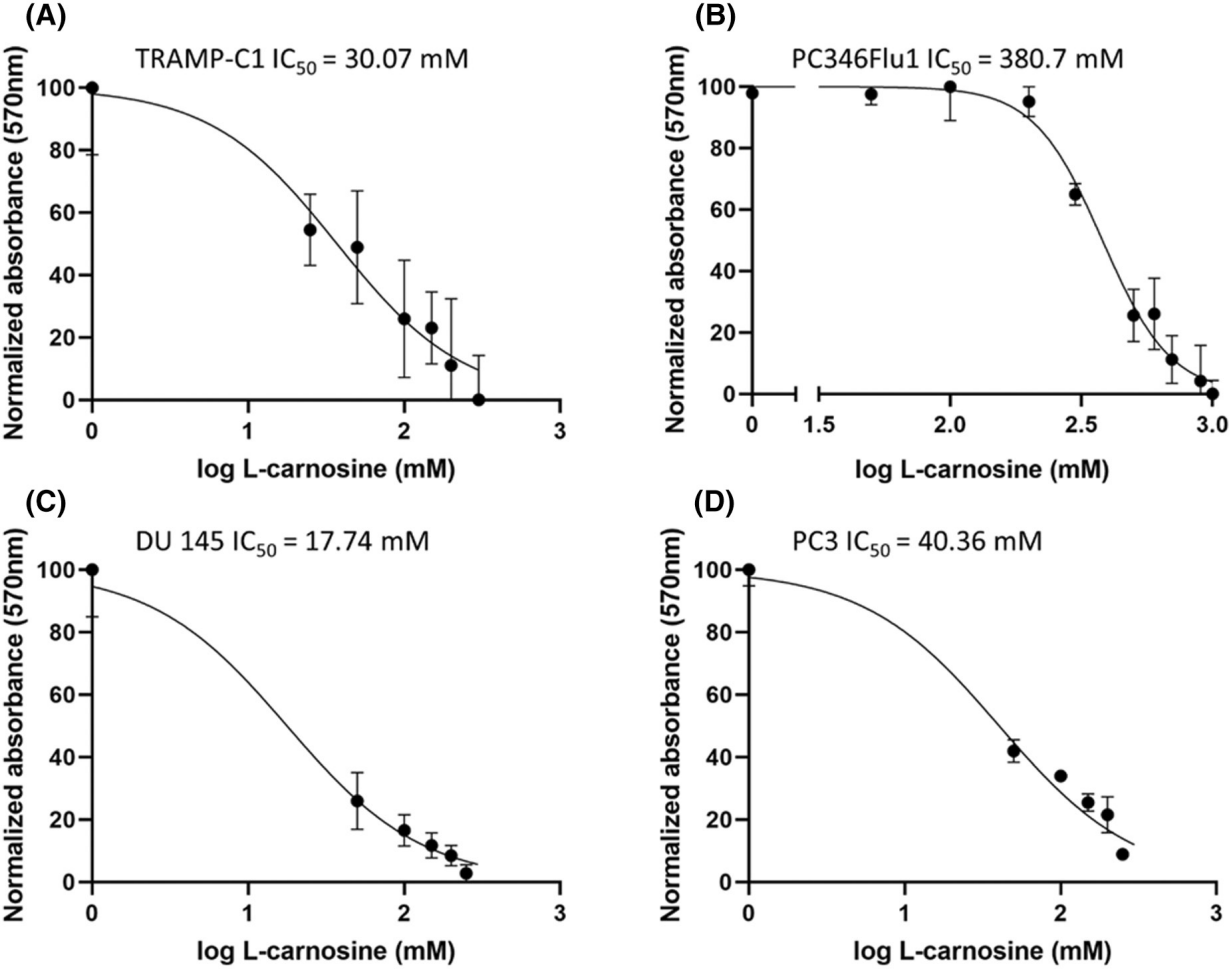
Carnosine affects mitochondrial functionality and slows down the proliferation of TRAMP‐C1 (A), PC346Flu1 (B), DU 145 (C) and PC3 (D) cells. MTT dose–response curves indicate that carnosine induces cell death and slows down proliferation in a dose‐dependent manner with IC_50_ of <50 mM in all cell lines besides PC346Flu1, which has an IC_50_ of 380.7 mM. Data points represent averages of three biological replicates.

MTT assay results reveal that carnosine affects the viability of TRAMP‐C1, PC346Flu1, DU 145 and PC3 cells (Figure 1), with the dose–response curve revealing the IC_50_ for TRAMP‐C1 (A), DU 145 (C) and PC3 (D) cell lines to be below 50 mM and for PC346Flu1 (B) cell line to be 380.7 mM.

**FIGURE 2:**
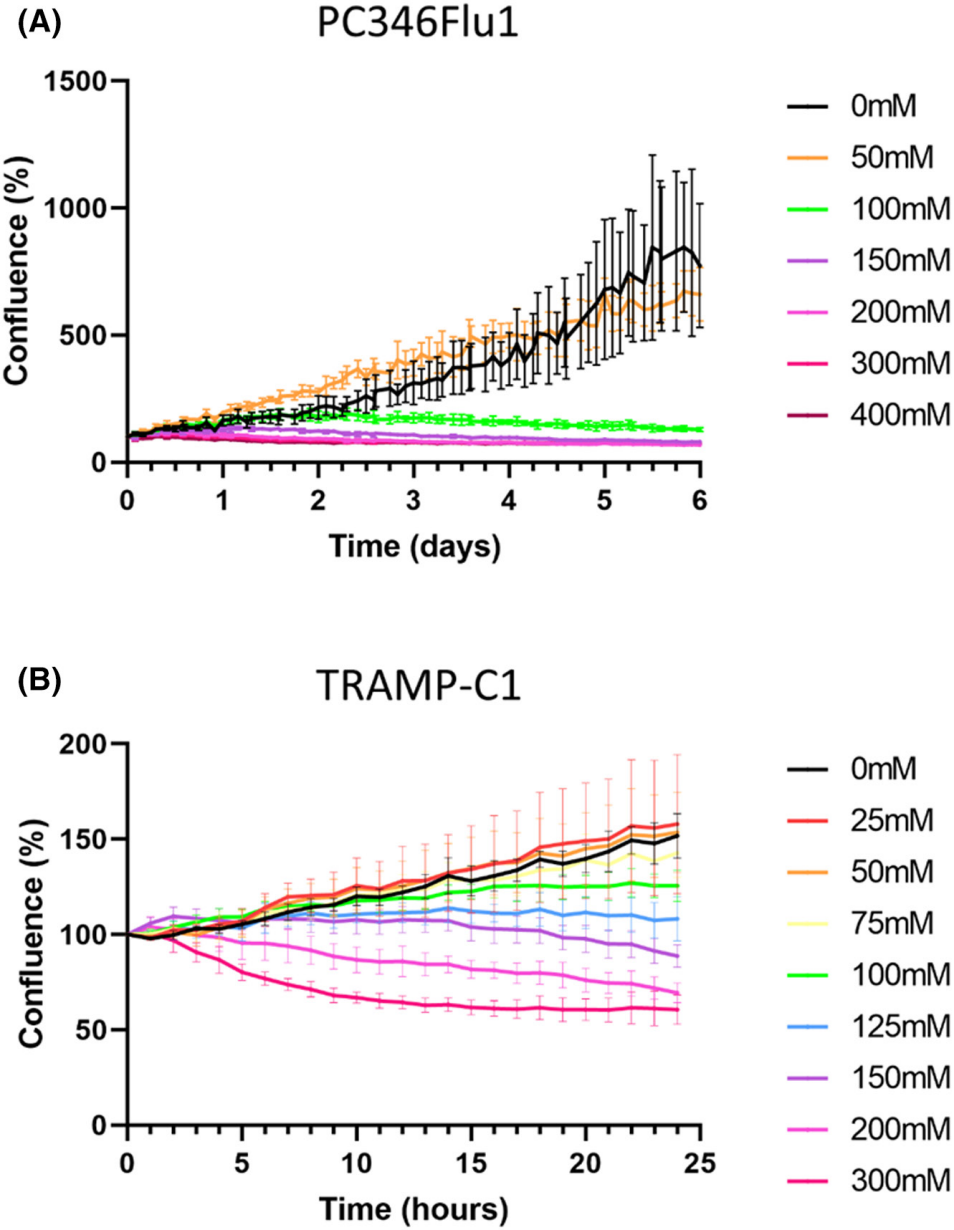
Live‐cell imaging analysis shows that carnosine affects the proliferation of both PC346Flu1 (A) and TRAMP‐C1 (B) cells. Carnosine inhibited the growth of each cell line in a dose‐ and time‐dependent manner. The maximum effect on proliferation was observed at a carnosine concentration of 300 mM. Data points represent averages of five technical replicates.

To further examine the anti‐cancer properties of carnosine, the effect of carnosine on the proliferation of both PC346Flu1 and TRAMP‐C1 cells was examined using the Incucyte Live‐Cell Analysis System. Figure 2 reveals that growth suppression was observed only hours after the addition of carnosine to the cells, with both cell lines showing a similar trend. Growth suppression occurred at a faster rate when the carnosine concentration was higher. Cells were clearly affected after 24 h of exposure to 100 mM and over of carnosine for TRAMP‐C1 and 72 h for PC346Flu1.

**FIGURE 3:**
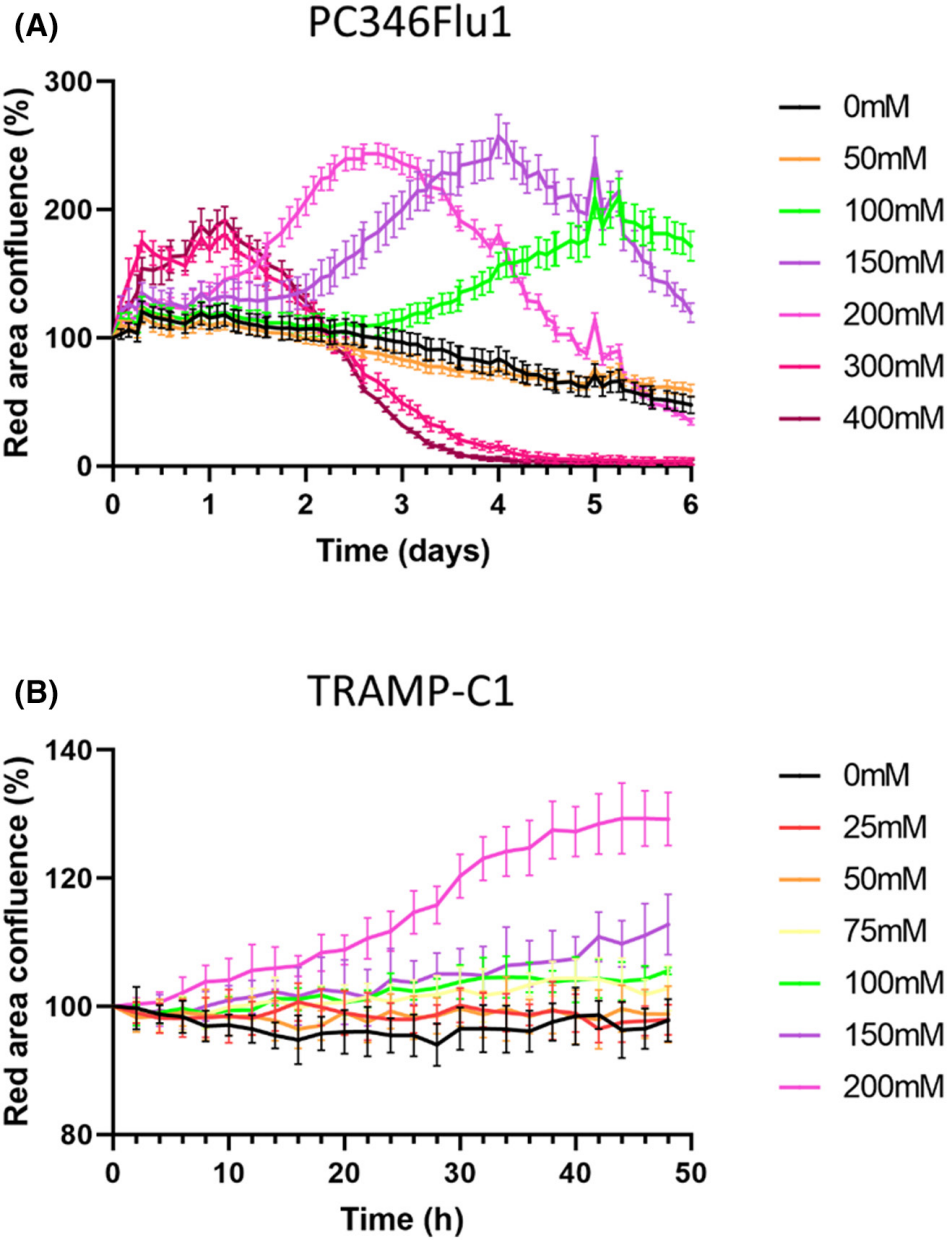
Live‐cell cytotoxicity analysis shows that carnosine induces the death of PC346Flu1 (A) and TRAMP‐C1 (B) cells. Carnosine inhibited cell growth in a dose‐ and time‐dependent manner due to the relatively different growth rates of the cells studied. The higher the dose of carnosine, the shorter the time required to reach cell death over 6 days for PC346Flu1 cells and over 48 h for TRAMP‐C1 cells. The maximum effect was shown starting from carnosine concentration of 300 mM for PC346Flu1 cells and 200 mM for TRAMP‐C1 cells. Data points represent averages of five technical replicates.

To further examine cell death induced by carnosine in real time we utilized phase contrast imaging of carnosine‐treated cells in combination with Incucyte Cytotox Red Dye (Figure 3). This method helped to provide an alternative assay for measuring cell death, with dead cells being fluorescently labelled due to the loss of their membrane integrity. Whilst an MTT assay was initially performed to measure cell viability, this assay is limited as it relies on the mitochondrial conversion of MTT to an insoluble formazan dye and is more a mark of mitochondrial activity as opposed to a true measure of cell death.

**FIGURE 4:**
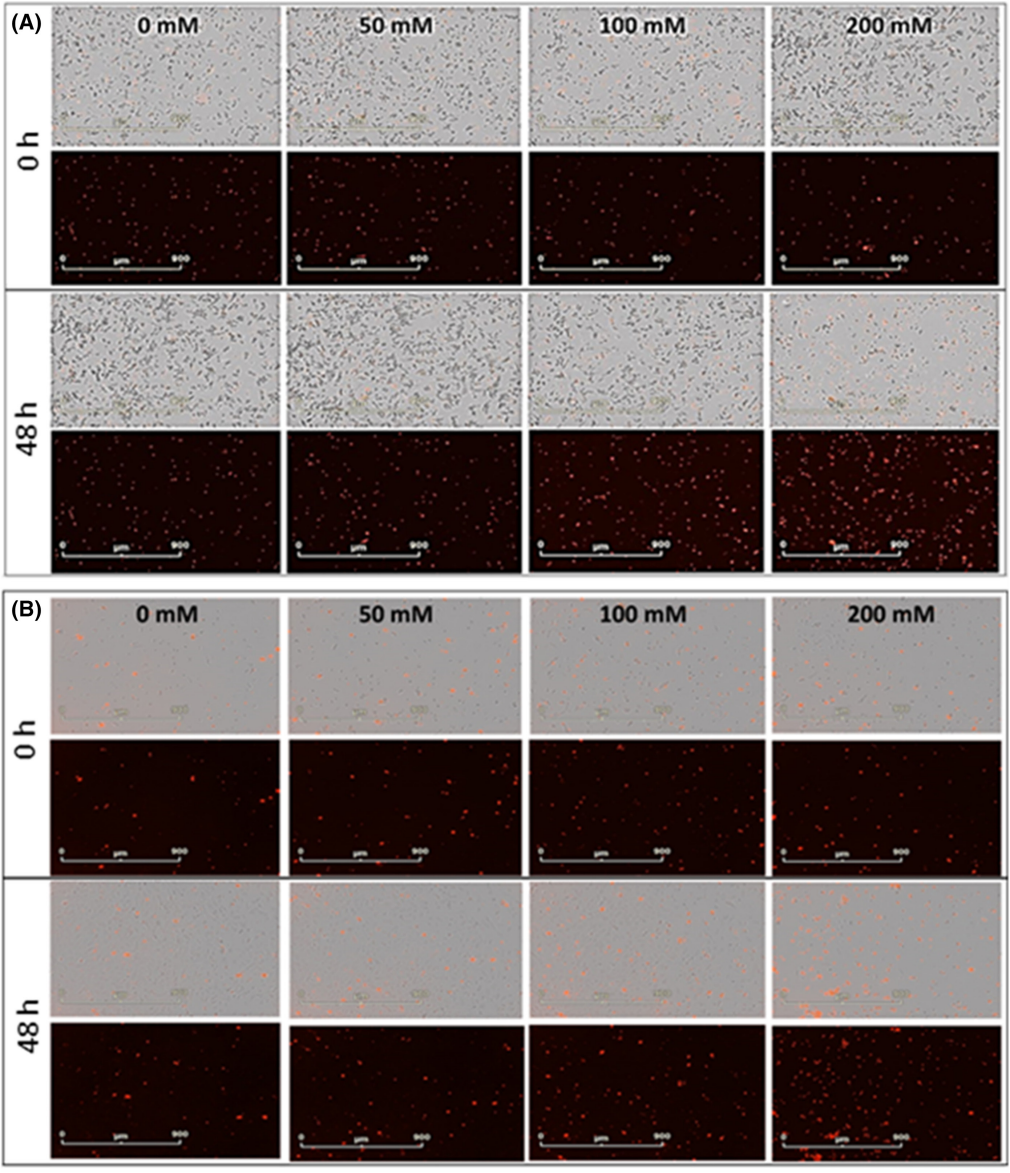
Live‐cell images of cytotoxicity upon carnosine exposure after 48 h for PC346Flu1 (A) and TRAMP‐C1 (B) cells. Cells were treated with different carnosine concentrations in the presence of Incucyte Cytotox Red Dye (250 nM), which enters dead cells after membrane integrity is lost and binds to DNA to emit red fluorescence. Carnosine‐induced cell death was observed from a concentration of 100 mM upwards. Images for both cell lines are displayed using the phase and red channels (top) and the red channel alone (bottom).

The Incucyte Cytotox Red Dye assay revealed that the number of dying cells increased in a dose‐dependent manner for both cell lines. The analysis of phase images and the red channel enabled real‐time evaluation of cell membrane integrity and cell death in response to carnosine exposure after 48 h (some example images are given in Figure 4), with red fluorescence being shown to increase after 48 h of carnosine exposure for both the PC346Flu1 (Figure 4A) and TRAMP‐C1 (Figure 4B) cell lines.

### 3.2 Carnosine treatment leads to a reduction of RS levels and high dose treatment leads to induction of SIRT3 expression

#### 3.2.1 Carnosine treatment leads to a reduction in RS levels

**FIGURE 5:**
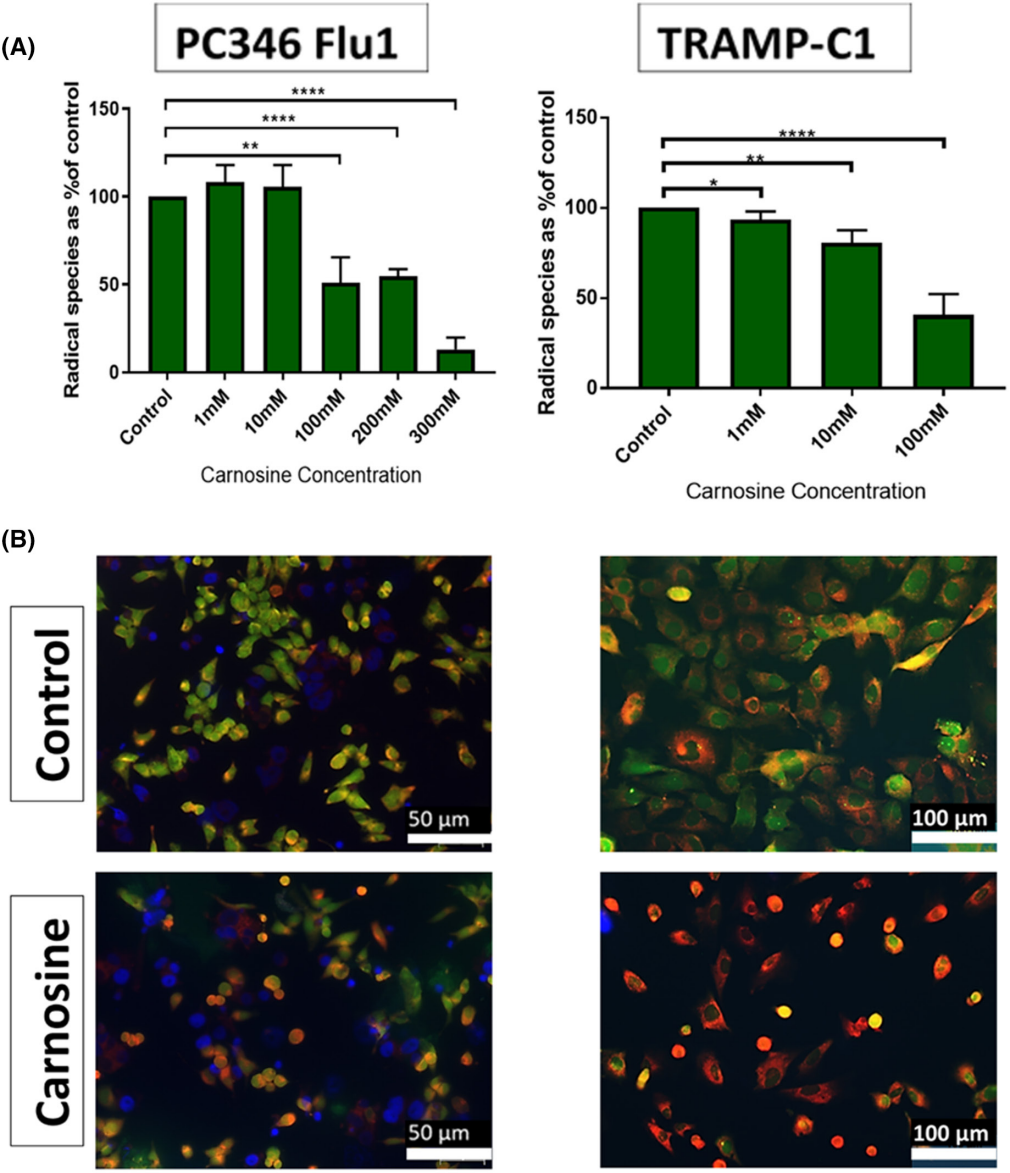
Carnosine reduces RS levels in PC346Flu1 and TRAMP‐C1 cells. (A) Radical species scavenging reveals that carnosine reduces radical species in a dose‐dependent manner (*n* = 3). (B) Treatment of PC346Flu1 cells with 100 mM resulted in a reduction of RS levels from 3 h post‐treatment. Mitochondria are stained red with MitoMark Red I; RS are indicated by green fluorescence; nuclei are stained blue by DAPI. **p < 0.01; ****p < 0.0001 as determined by Student's *t*‐test.

The cytotoxic effect of carnosine treatment on PC346Flu1 cells and TRAMP‐C1 cells as detected by the MTT assay indicated a direct effect of carnosine on mitochondria. We therefore set out to determine if there were any changes in the RS levels in those cells after carnosine treatment. Results showed a significant reduction of RS as the carnosine concentration increased above 100 mM. The formation of RS was visualized by means of the H_2_DCFDA probe, which confirmed the decrease in RS within the mitochondria (Figure 5B).

#### 3.2.2 Carnosine induces the expression of SIRT3 in TRAMP‐C1 and PC346Flu1 cells

**FIGURE 6:**
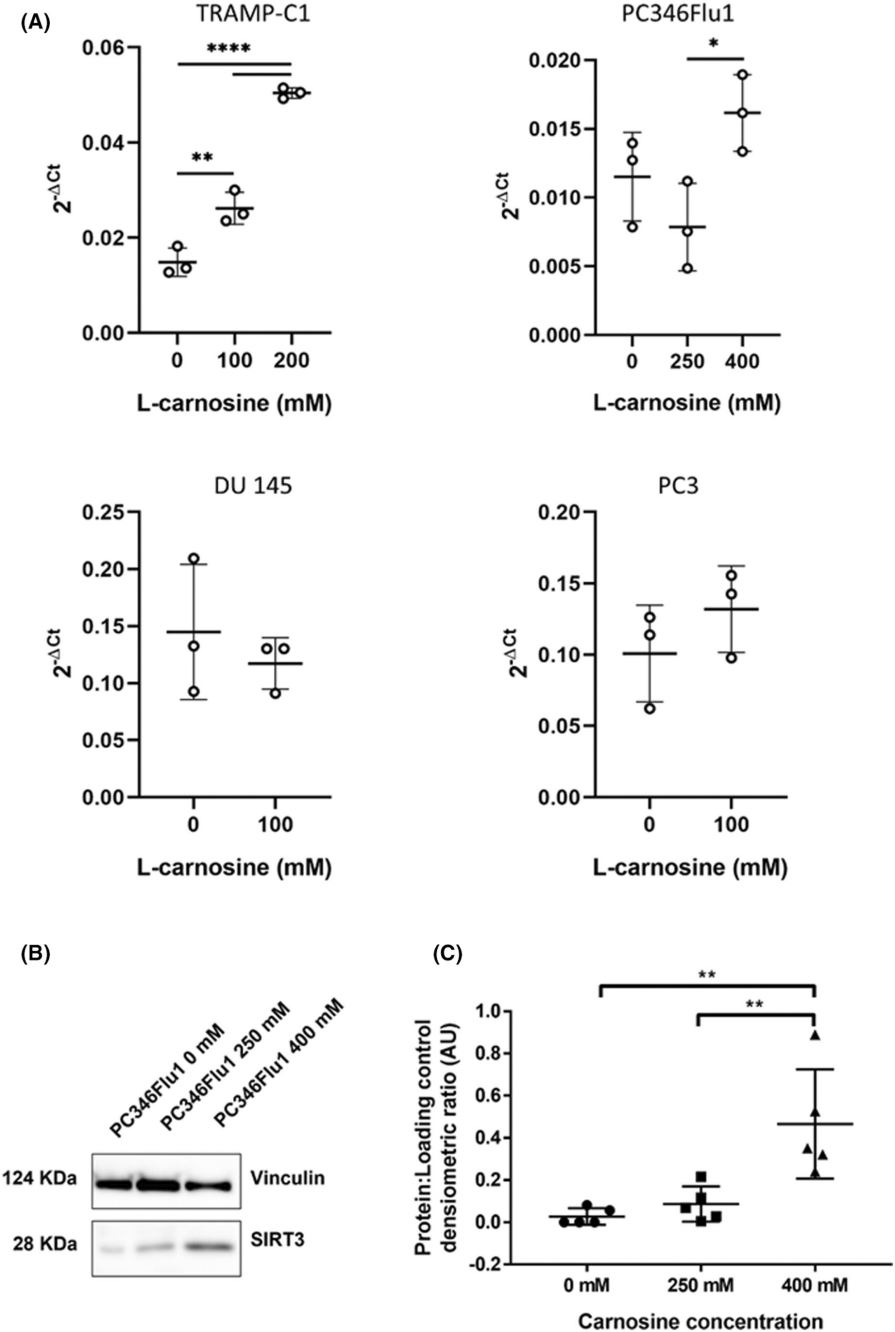
Carnosine increases SIRT3 levels in TRAMP‐C1 and PC346Flu1 cells. (A) RT‐qPCR reveals a significant increase in SIRT3 mRNA levels in TRAMP‐C1 and PC346Flu1 cells after 24 h of treatment with 100 mM, 200 mM (TRAMP‐C1), 250 mM and 400 mM (PC346Flu1) of carnosine. No significant increase of SIRT3 mRNA levels was observed in DU 145 and PC3. Data points represent three biological replicates. (B) Western blotting revealed that carnosine induced expression of SIRT3 protein in PC346Flu1 cells. (C) Quantification of SIRT3 protein levels as detected by western blot. Data points represent five biological replicates. *p < 0.05; **p < 0.01 as determined by a one‐way anova.

Due to the reduction of RS after carnosine treatment it was decided to examine whether carnosine resulted in any changes to sirtuin 3 (SIRT3), a protein known to be a master regulator of RS. Previous research has shown that SIRT3 plays a key role in RS reduction, with SIRT3 being involved in mitochondrial respiration and downstream events that reduce RS production.^20^, ^21^ When PC346Flu1 cells were treated with carnosine (400 mM) for 24 h, levels of SIRT3 RNA and protein increased significantly (Figure 6A–C), a finding that corroborates the reduction in RS shown in carnosine‐treated cells (Figure 5A). SIRT3 mRNA levels were also significantly increased after treatment of TRAMP‐C1 cells with 100 and 200 mM of carnosine for 24 h, but no differences in mRNA expression were observed in DU 145 nor PC3 cells (Figure 6A).

### 3.3 Carnosine affects the ATP production of PC346Flu1 and TRAMP‐C1 cells by affecting both the mitochondrial OXPHOS and glycolysis pathways in a dose‐dependent manner

**FIGURE 7:**
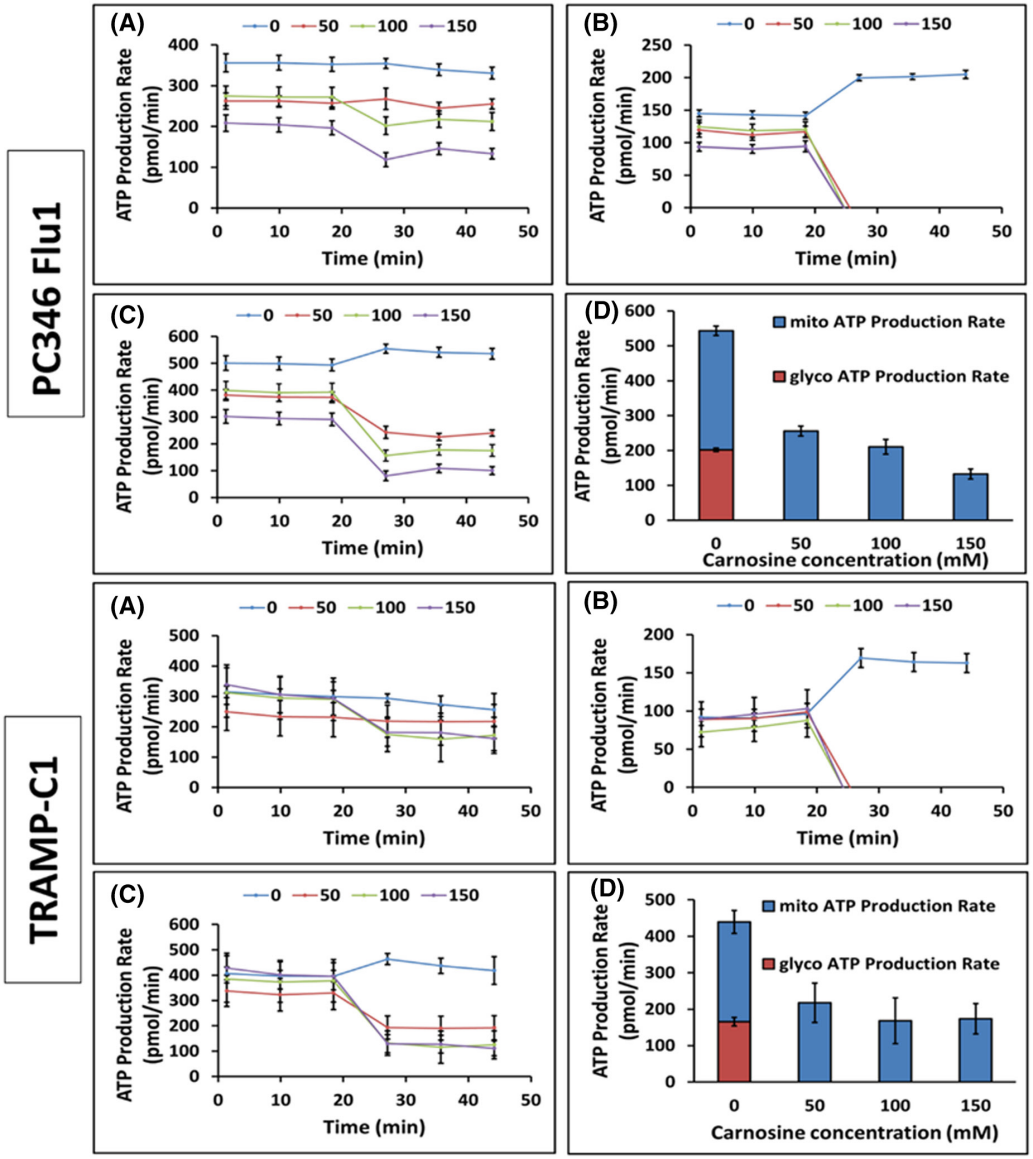
Seahorse flux analysis of cells treated with carnosine. (A) Mitochondrial ATP production rate data (basal and induced rates). (B) Glycolysis ATP production rate data (basal and induced rates). (C) Total ATP production rate data (basal and induced rates). (D) Induced rates average of ATP production (*n* = 3).

The Agilent Seahorse XF Real‐Time ATP Rate Assay is designed to measure total ATP production rates in living cells and to distinguish between the two main metabolic pathways responsible for ATP production in mammalian cells. Fractions of ATP were calculated as being generated by either mitochondrial oxidative phosphorylation (OXPHOS) or glycolysis. The assay was applied on each cell line (PC346Flu1 80,000 cells per well and TRAMP‐C1 40,000 cells per well). Figure 7 shows that carnosine treatment has an immediate effect on ATP production via the glycolytic pathway as an inhibition was observed within 20 min of carnosine treatment. Mitochondria were still producing ATP even after treatment with the highest concentration of carnosine (150 mM); however, the indicated gradual reduction of OXPHOS was shown with increasing carnosine concentrations. These results highlight that carnosine reduces the overall ATP production of both the PC346Flu1 and TRAMP‐C1 cell lines.

### 3.4 Continuous dosing of carnosine is necessary to affect spheroid growth

**FIGURE 8:**
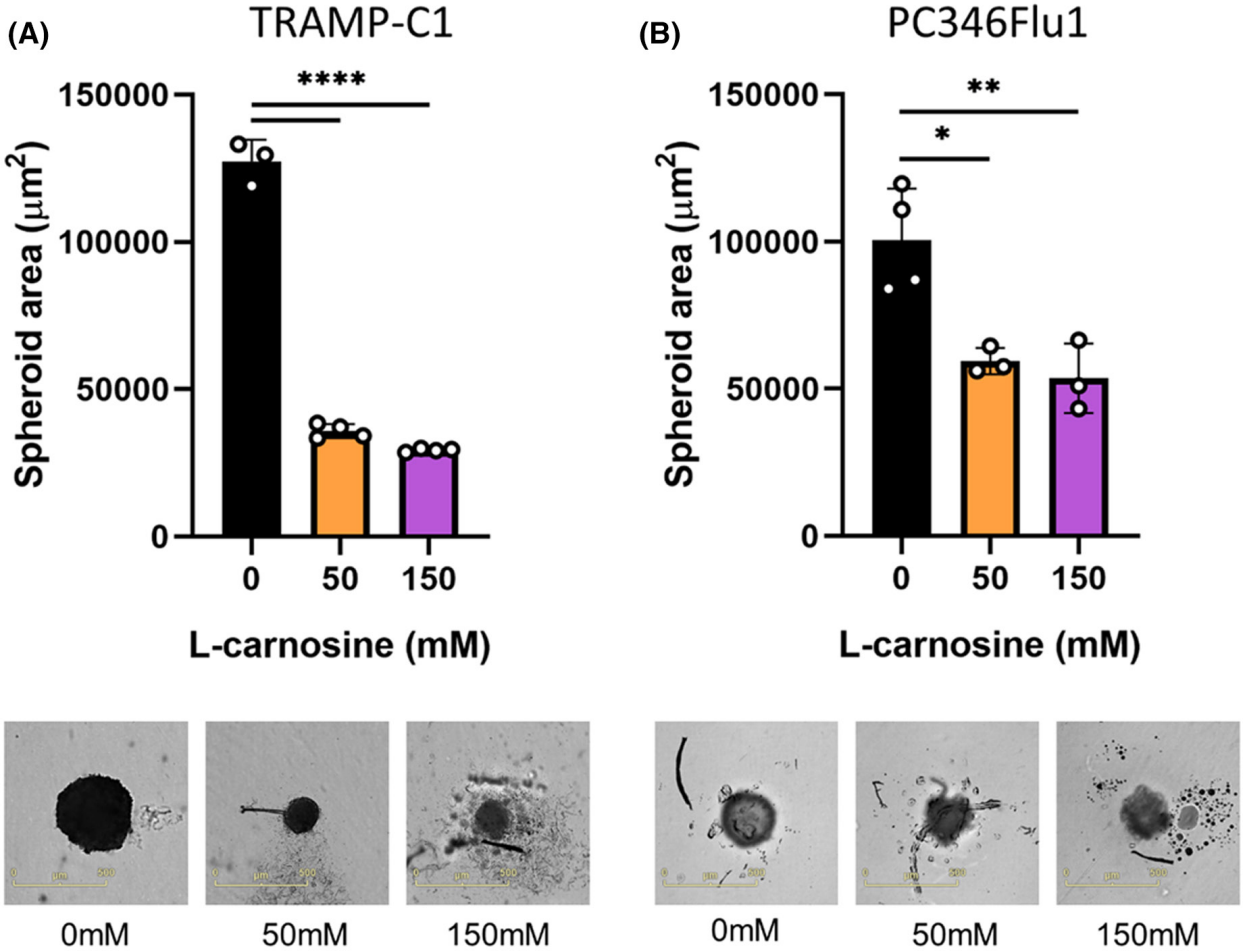
Effect of sustained carnosine treatment on PC346Flu1 (A) and TRAMP‐C1 (B) spheroids. Carnosine was added every other day different concentrations ranging from 0 to 150 mM, with images being collected after 7 days. Spheroid integrity was affected with carnosine concentrations of 50 mM and above, with a significant difference being observed regarding spheroid area (μm^2^). Data points represent technical replicates. *p < 0.05; **p < 0.01; ****p < 0.0001 as determined by a one‐way anova.

The effect of carnosine on cells grown as a monolayer is likely to be different from cells growing tightly together, as is the case in tumours. This tight structure prevents the penetration of carnosine into the tumour core, reducing the levels of carnosine exposure for cells located in the core. Therefore, to recapitulate these features and get one step closer to an in vivo growing tumour, cells were grown in 3D spheroids. Not surprisingly we showed that carnosine needed to be added more than once to influence 3D cultures. When carnosine was added every other day, mimicking sustained release/long‐term carnosine treatment, the growth of PC346Flu1 and TRAMP‐C1 was significantly reduced (Figure 8). These experiments reflect the importance of the need for a constant sustained release of carnosine for the treatment of cancer cells. The change in the morphology of single spheroids was monitored using the Incucyte Spheroid Analysis Software Module over 7 days. Single spheroids were generated and then treated every 48 h with carnosine.

## 4 DISCUSSION

Treatments for organ‐confined prostate cancer are not cancer‐specific and are commonly accompanied by side effects, including urinary incontinence and erectile dysfunction. Moreover, subsequent additional surgical interventions following biochemical recurrence are either limited or are affected by the scarring present in the surrounding tissue. Carnosine is a naturally occurring dipeptide which has been shown to specifically affect cancer cells over healthy cells.^11^, ^15^ The effect of carnosine has been studied on many cancers cell types,^10^ but never on prostate cancer cells. We therefore set out to investigate the potential anti‐cancer properties of carnosine on TRAMP‐C1 cells, mouse prostate cancer cells, and PC346Flu1, a primary human androgen resistant prostate cancer cell line. MTT data show that carnosine affects mitochondrial function and reduces cell viability by 50% at carnosine concentrations lower than 50 mM for TRAMP‐C1 and just under 400 mM for PC346Flu1. This was also accompanied by decreased levels of RS, with data being consistent with previously reported findings.^22^ MTTs were also performed for two metastatic prostate cancer cell lines, DU 145 and PC3 (Figure 1C,D), also showing IC_50_ values below 50 mM, similar to TRAMP‐C1.

SIRT3 has been identified as a key regulator of RS and oxygen metabolism balance^23^, ^24^ and was therefore investigated to see whether the decrease in RS after carnosine treatment could be in part due to an increase in SIRT3. SIRT3 expression was examined in the PC346Flu1 cell line after 24 h of carnosine exposure and shown to be significantly increased at both mRNA transcript and protein level, correlating with the reduction in RS levels observed. Of note, SIRT3 mRNA levels were not significantly increased in the two metastatic cell lines tested, DU 145 and PC3, following carnosine treatment, and this was also true for immortalized prostate cells (Figure S2). Importantly, SIRT3 expression has previously been shown to be downregulated in prostate cancer and knock‐in of *SIRT3* in prostate cancer cells has been shown to have anti‐tumour effects.^25^ SIRT3 has also been shown to reduce the migration and epithelial‐to‐mesenchymal transition of prostate cancer cells.^26^ As such, in addition to direct effects of carnosine via free radical species scavenging, SIRT3 upregulation following carnosine exposure suggests additional potential SIRT3‐mediated anti‐cancer actions. This would help to prevent the spread of localized prostate cancer, thus facilitating more effective treatment.

The effect of carnosine on the viability and proliferation of PC346Flu1 and TRAMP‐C1 cells was further demonstrated by live‐cell imaging, with a decrease of live cell numbers and an increase in the number of dying cells occurring in a dose‐dependent manner. This is the first time that the effect of carnosine has been investigated on prostate cancer cells. However, the first potential antineoplastic effect for carnosine was proposed nearly four decades ago.^27^ Sarcoma tumour cells were implanted subcutaneously in vivo, and carnosine was administrated near the implantation site every other day. As a result, both tumour growth and mortality were reduced.^28^ Since then, other publications have supported the selective effect of carnosine in suppressing transformed and neoplastic cells.^29^, ^30^, ^31^ However, this selective effect is likely due to the preferential use of the glycolytic pathway by these cells rather than their transformed nature. Indeed, immortalized cells, which share the same metabolic shift towards glycolysis as transformed and neoplastic cells, are also affected by carnosine in a dose‐dependent manner (Figure S1). Daily intraperitoneal injection of 1 M carnosine resulted in the delay of aggressive tumour growth of NIH3T3 fibroblasts, transfected with human epidermal growth factor receptor 2 (HER2/neu) in nude mice.^29^ Moreover, a significant advantage of carnosine administration is its effect on healthy cells, which has been reported through increased cell viability.^15^ We have also conducted some toxicity experiments and shown that carnosine is not toxic to PBMC nor red blood cells (Figures S3 and S4).

Healthy cells utilize OXPHOS to produce energy in normoxic conditions. By contrast, cancer cells mainly use the glycolysis pathway, even in the presence of oxygen, to obtain both ATP and the necessary anabolic precursors for rapid proliferation, known as the Warburg effect.^32^ We have shown here that the primary mechanism of action of carnosine in prostate cancer cells is to inhibit glycolysis and decrease ATP production.^33^ A mechanism whereby the activity of the glycolytic enzyme, glyoxalase, regulating protein glycation, cell apoptosis, gene expression, redox biology, and metastasis is modulated by carnosine was hypothesized in a previous review.^34^ Unlike normal cells, prostate cells have a unique metabolism characterized by an incomplete Krebs cycle which terminates early with the secretion of citrate. This tricarboxylic acid (TCA) cycle truncation is caused by the excess of zinc present in the cells which prevents the catalysis of the oxidation of citrate into isocitrate by mitochondrial aconitase (ACO2), the first step of the Krebs cycle. During the transformation of normal prostate cells into malignant cells, the TCA cycle is restored, and prostate cancer cells use the OXPHOS pathway to obtain most of their ATP. However, progression to advanced prostate cancer and resistance to androgen deprivation therapy is associated with a metabolic switch to glycolysis. Both TRAMP‐C1 and PC346Flu1 cells grow without the presence of androgen and, whilst they appear to use both OXPHOS and glycolysis, results obtained from the Seahorse experiments show that carnosine affected the growth and viability of these cells mainly by affecting the glycolytic pathway. However, whilst a single dose was sufficient to influence the cells grown as a monolayer, we showed that repeated doses were required to have the same effect on cells grown as spheroids, indicating that future use of carnosine for the treatment of localized prostate cancer will need either repeated carnosine administration, or else delivery in a sustained manner.

Consequently, carnosine and associated analogs hold therapeutic benefit in a primary prostate cancer setting. We are now investigating this in a preclinical model of prostate cancer.

## AUTHOR CONTRIBUTIONS

**K. Habra:** Data curation (equal); methodology (equal). **J. R. D. Pearson:** Data curation (supporting); formal analysis (supporting); investigation (supporting); methodology (supporting). **P. Le Vu:** Methodology (supporting). **C. Puig‐Saenz:** Data curation (equal); formal analysis (equal); investigation (equal); visualization (equal); writing – review and editing (equal). **M. J. Cripps:** Methodology (supporting). **M. A. Khan:** Resources (equal). **M. D. Turner:** Conceptualization (equal); writing – review and editing (equal). **C. Sale:** Conceptualization (equal); writing – review and editing (equal). **S. E. B. McArdle:** Conceptualization (equal); data curation (equal); project administration (equal).

## FUNDING INFORMATION

This study was supported by the John and Lucille van Geest Foundation and Nottingham Trent University Health and Wellbeing Seedcorn Award.

## CONFLICT OF INTEREST STATEMENT

C.S. has received funding to support a PhD studentship relating to the effects of carnosine on cardiac function from Natural Alternatives International; a company formulating and manufacturing customized nutritional supplements, including CarnoSyn beta‐alanine. The same company has also provided C.S. with supplements for other studies free of charge, have contributed to the payment of open access publication charges for some manuscripts on beta‐alanine supplementation and have paid an honorarium to produce material for a blog relating to beta‐alanine supplementation and carnosine. None of this work, however, relates directly to the study reported herein.

## Supporting information


Figure S1.

Figure S2.

Figure S3.

Figure S4.

## Data Availability

N/A.
