# Supplementary figures and images for: Anticancer actions of carnosine in cellular models of prostate cancer
Source: J Cell Mol Med. 2023 Nov 29;28(2):e18061. doi: 10.1111/jcmm.18061 (PMC10826443; doi:10.1111/jcmm.18061)

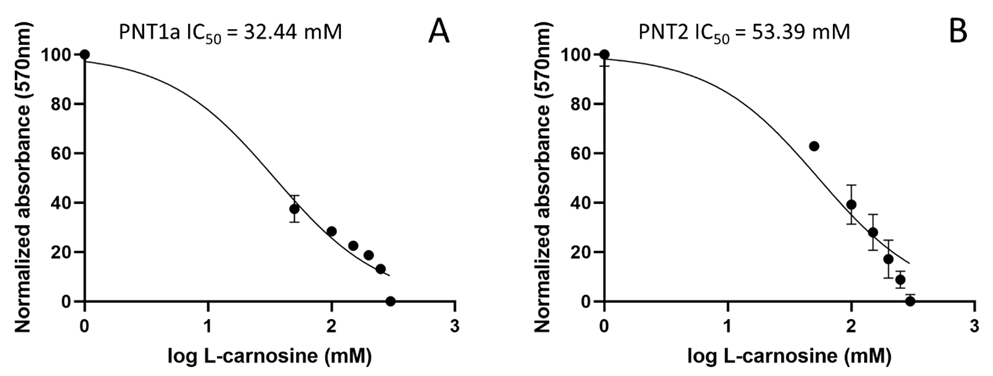

Supplement: Supplementary file 1 — Figure S1. [file JCMM-28-e18061-s003.tif]

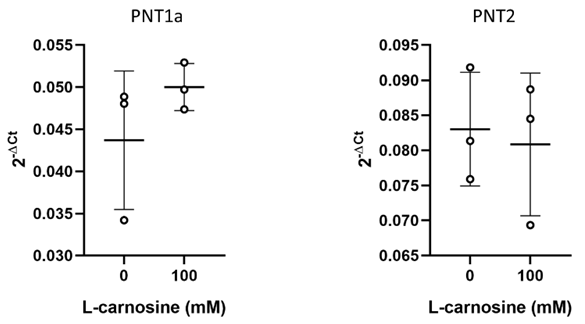

Supplement: Supplementary file 2 — Figure S2. [file JCMM-28-e18061-s004.tif]

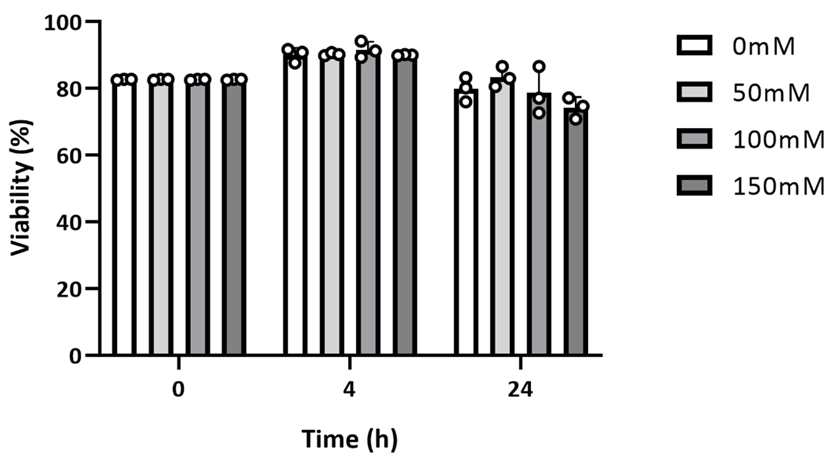

Supplement: Supplementary file 3 — Figure S3. [file JCMM-28-e18061-s002.tif]

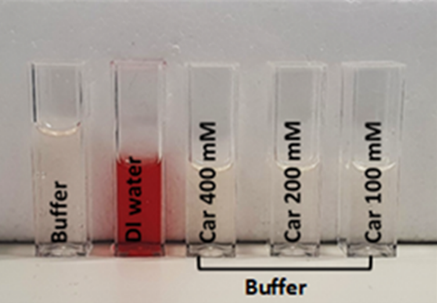

Supplement: Supplementary file 4 — Figure S4. [file JCMM-28-e18061-s001.tif]
